# Relationships of Hyperhomocysteinemia and Hyperuricemia With Metabolic Syndrome and Renal Function in Chinese Centenarians

**DOI:** 10.3389/fendo.2018.00502

**Published:** 2018-09-11

**Authors:** Shihui Fu, Yao Yao, Yali Zhao, Fuxin Luan

**Affiliations:** ^1^Department of Geriatric Cardiology, Chinese People's Liberation Army General Hospital, Beijing, China; ^2^Department of Cardiology and Hainan Branch, Chinese People's Liberation Army General Hospital, Beijing, China; ^3^Institute of Geriatrics, Chinese People's Liberation Army General Hospital, Beijing, China; ^4^Beijing Key Laboratory of Geriatrics, Chinese People's Liberation Army General Hospital, Beijing, China; ^5^Central Laboratory, Hainan Branch of Chinese People's Liberation Army General Hospital, Sanya, China

**Keywords:** Chinese centenarians, hyperhomocysteinemia, hyperuricemia, metabolic syndrome, renal function

## Abstract

As the first time worldwide, this study aimed to investigate the relationships of hyperhomocysteinemia and hyperuricemia with metabolic syndrome (MetS) and renal function in Chinese centenarians. The China Hainan Centenarian Cohort Study was performed in 18 cities and counties of the Hainan Province. Home interview, physical examination, and blood analysis were performed on 808 centenarians following standard procedures. All centenarians had a median age of 102 (100–115) years. Prevalence of hyperhomocysteinemia and hyperuricemia was 91.6% (740 centenarians) and 28.5% (230 centenarians), respectively. The MetS was present in 117 centenarians (14.5%). In simple correlation analyses, hyperhomocysteinemia and hyperuricemia were significantly correlated with MetS and glomerular filtration rate (GFR) < 60 ml/min/1.73 m^2^ (*P* < 0.05 for all). Serum homocysteine levels were significantly correlated with GFR, waist circumference (WC), and triglyceride levels, while serum uric acid levels were significantly correlated with these variables plus high-density lipoprotein cholesterol (HDL-C) levels (*P* < 0.05 for all). In logistic regression analyses, hyperhomocysteinemia and hyperuricemia were significantly associated with MetS and GFR < 60 ml/min/1.73 m^2^ (*P* < 0.05 for all). In linear regression analyses, serum homocysteine levels were significantly associated with GFR, WC, and triglyceride, while serum uric acid levels were significantly associated with these variables plus HDL-C (*P* < 0.05 for all). Both hyperhomocysteinemia and hyperuricemia had important relationships with MetS and renal function in Chinese centenarians. Hyperuricemia and hyperhomocysteinemia that could help identify, while also affecting, the development of MetS and renal function may unfold complex relationships between MetS, renal function, and cardiovascular risk and provide effective prevention strategies for these conditions.

## Introduction

As a complex pathophysiological entity, metabolic syndrome (MetS) comprises a number of metabolic abnormalities and is characterized by insulin resistance (1). The MetS has been estimated to occur in between 20 and 25% of the adult population and suggested as a leading cause of cardiovascular diseases and mortality (2–4). Homocysteine, a thiol-containing amino acid produced during the conversion of methionine to cysteine, has been found to correlate with insulin resistance and MetS in previous studies (5–8). However, it is being debated whether these correlations exist or do not persist, thus, making it a contentious issue in the literature (9–11). Meanwhile, there is growing interest in the relationship between hyperuricemia and MetS, which has been investigated previously in different populations, but with conflicting results (12, 13). In addition to MetS, renal function decline is also an important worldwide public health problem and has gained much attention in recent years (14). Epidemiological studies have realized a relationship between hyperuricemia and renal function (15–17). However, these studies have shown disputable results and it is, therefore, difficult to draw definite conclusions (18). Meanwhile, studies have also reported that hyperhomocysteinemia is an important indicator for renal function (19, 20). There is also, however, controversial and limited evidence about hyperhomocysteinemia and renal function in different populations (21).

The centenarians have been suggested to have delayed or escaped onset and interaction of age-related illnesses, such as hyperuricemia, hyperhomocysteinemia, MetS, and renal function decline (22). Some centenarians may experience delayed onset of age-related illnesses (delayers), while others may not succumb to any age-related illnesses (escapers) (23). Thus, the centenarians may represent a prototype of successful aging (24). However, this trend is still under scientific debate (25). More importantly, what is this model of successful aging? Studies analyzing this model in the centenarians could provide valuable information for early promotion of successful aging and prevention of age-related diseases. As a possible part of this model, whether the relationships of hyperuricemia and hyperhomocysteinemia with MetS and renal function decline exist in the aging process of centenarians is still unclear and needs further studies. Most of the current understandings about these relationships are derived from studies on general adults and the elderly population from Caucasians of European origin (26). It is well recognized that age and ethnicity may have a remarkable effect on these relationships (27). Specifically, the senile population and Asian adults are recognized to be more susceptible to adverse effects of MetS (28). To our knowledge, hardly any studies have confirmed these relationships in centenarians, especially in China. It is interesting to analyze these relationships in Chinese centenarians. Hainan is an area where longevity is high, with the highest population density of centenarians in China. The China Hainan Centenarian Cohort Study (CHCCS), with a considerable sample size, provides a significant population-based sample of Chinese centenarians.

As it is the first in the world, the current study aims to investigate the relationships of hyperhomocysteinemia and hyperuricemia with MetS and renal function in Chinese centenarians.

## Methods

### Study population

As a population-based study, the CHCCS was carried out in 18 cities and counties of the Hainan Province, China. Its cohort profile has been described previously (29). It started in July 2014 and ended in December 2016. Based on the National Civil Registry, there were 1,002 centenarians (at least 100 years of age) identified by the Hainan Civil Affairs Bureau and surveyed in the current study. Age was ascertained from national identification cards. The following inclusion criteria were used to recruit study participants: (1) were 100 years or older; (2) had volunteered to participate in the study and provided written informed consent; and (3) were conscious and could cooperate to complete the home interview, physical examination, and blood analysis. The following were participant exclusion criteria: (1) personal identity information was not complete or identification cards showed an age < 100 years; and (2) refused to comply with the requirements of the study, including the collection of physical information or blood samples. There were 808 centenarians included in the final analysis. All centenarians had no certain malignancies and received no agent that obviously changed serum homocysteine and uric acid levels. The Ethics Committee of Hainan branch of Chinese People's Liberation Army General Hospital (Sanya, Hainan) has approved of the current study (Number: 301hn11201601), and all centenarians gave written informed consent upon their entry.

### Standard procedures

Home interview, physical examination, and blood analysis were performed following standard procedures (30). The research team included internists, geriatricians, cardiologists, endocrinologists, nephrologists, and nurses. Waist circumference (WC) was measured with a soft tape in the middle point of the lowest rib and iliac crest. After resting in supine position for 5 min, systolic and diastolic blood pressures (SBP and DBP) were measured twice on the right arm of the centenarians with a 1-min interval. The average of these measurements was used for the analysis. Samples of venous blood were drawn from the centenarians and transported in chilled bio-transport containers (4°C) to our Central Laboratory within 4 h. Serum concentrations of triglyceride, high-density lipoprotein cholesterol (HDL-C), low-density lipoprotein cholesterol (LDL-C), fasting blood glucose (FBG), and creatinine were tested using the enzymatic assays (Roche Products Ltd, Basel, Switzerland) on a fully automatic biochemical autoanalyzer (Cobas c702; Roche Products Ltd., Basel, Switzerland). All assays were performed by qualified technicians without knowledge of clinical data.

### Variable definitions

According to the worldwide consensus of the International Diabetes Federation (31), the centenarians with WC ≥85 cm in males and ≥80 cm in females along with any two or more of the following abnormalities were considered to have MetS: (1) SBP ≥130 mmHg or DBP ≥85 mmHg (or use of blood pressure-lowering agents); (2) FBG ≥5.6 mmol/L (or use of glucose-lowering agents); (3) triglyceride ≥1.7 mmol/L (or use of lipid-regulating agents); and (4) HDL-C < 1.0 mmol/L in males and < 1.3 mmol/L in females. Estimated glomerular filtration rate (GFR) was calculated using a modified version of the Modification of Diet in Renal Disease (MDRD) equation based on the data from Chinese population as follows: 175 × serum creatinine (mg/dL)^−1.234^ × age (year)^−0.179^ × 0.79 (if female) (32). Hyperhomocysteinemia was diagnosed as serum homocysteine levels above 15 umol/L. Hyperuricemia was diagnosed as serum uric acid levels above 420 umol/L in males or 350 umol/L in females.

### Statistical analyses

To describe the variables of Chinese centenarians, mean (standard deviation), median (interquartile range) and number (percentage) were applied for normally distributed continuous variables, non-normally distributed continuous variables, and categorical variables, respectively. Pearson's (normally distributed continuous variables) and Spearman's (non-normally distributed continuous variables and categorical variables) correlations were applied to evaluate their simple correlations with serum homocysteine (hyperhomocysteinemia) and uric acid (hyperuricemia) levels. Logistic regression analyses were applied to evaluate the associations of hyperhomocysteinemia and hyperuricemia with GFR < 60 ml/min/1.73 m^2^ after adjusting for age, sex, WC, SBP, DBP, triglyceride, HDL-C, LDL-C, and FBG, and with MetS after adjusting for age and sex. With the same adjustment, linear regression analyses were applied to evaluate the associations of serum homocysteine and uric acid levels with GFR and all features of MetS. Two-tailed tests were adopted throughout, considering significant *P* < 0.05. The Statistical Package for Social Science (SPSS) version 17 was applied to carry out all statistical analyses (SPSS Inc., Chicago, IL, United States).

## Results

As shown in Table 1, the current study included 808 centenarians with a median age of 102 (100-115) years. It consisted of 155 men (19.2%) and 653 women (80.8%). Prevalence of hyperhomocysteinemia and hyperuricemia was 91.6% (740 centenarians) and 28.5% (230 centenarians), respectively. The MetS was presented in 117 centenarians (14.5%). In simple correlation analyses, hyperhomocysteinemia and hyperuricemia were significantly correlated with MetS and GFR < 60 ml/min/1.73 m^2^ (*P* < 0.05 for all).^.^ Serum homocysteine levels were significantly correlated with GFR, WC, and triglyceride levels, while serum uric acid levels were significantly correlated with these variables plus HDL-C levels (*P* < 0.05 for all). In logistic regression analyses (Table 2), hyperhomocysteinemia and hyperuricemia were significantly associated with MetS and GFR < 60 ml/min/1.73 m^2^ (*P* < 0.05 for all). In linear regression analyses (Table 3), serum homocysteine levels were significantly associated with GFR, WC, and triglyceride, while serum uric acid levels were significantly associated with these variables plus HDL-C (*P* < 0.05 for all).

**Table 1:** Variables of Chinese centenarians and their correlations with serum homocysteine (hyperhomocysteinemia) and uric acid (hyperuricemia) levels.

**Variables**	**Description**	***r*-value**	***P*-value**	***r*-value**	***P*-value**
Age (year)	102 (101–104)	0.028^a^	0.422^a^	−0.060^b^	0.089^b^
Males (%)	155 (19.2)	−0.137^a^	<0.001^a^	−0.256^b^	<0.001^b^
Hyperhomocysteinemia (%)	740 (91.6)	1.000^c^	<0.001^c^	0.112^d^	0.001^d^
Homocysteine (umol/L)	23.60 (18.73–29.80)	1.000^a^	<0.001^a^	0.267^b^	<0.001^b^
Hyperuricemia (%)	230 (28.5)	0.112^c^	0.001^c^	1.000^d^	<0.001^d^
Uric acid (umol/L)	316.50 (260.25–382.75)	0.267^a^	<0.001^a^	1.000^b^	<0.001^b^
GFR < 60 ml/min/1.73 m^2^ (%)	344 (42.6)	0.198^c^	<0.001^c^	0.333^d^	<0.001^d^
GFR (ml/min/1.73 m^2^)	63.11 (52.84–73.93)	−0.436^a^	<0.001^a^	−0.525^b^	<0.001^b^
MetS (%)	117 (14.5)	0.074^c^	0.035^c^	0.083^d^	0.018^d^
WC (cm)	75 (70–80)	0.126^a^	<0.001^a^	0.193^b^	<0.001^b^
SBP (mmHg)	151 (136–170)	0.062^a^	0.079^a^	−0.033^b^	0.344^b^
DBP (mmHg)	76 (67–84)	0.024^a^	0.489^a^	−0.056^b^	0.110^b^
Triglyceride (mmol/L)	1.04 (0.80–1.40)	0.073^a^	0.039^a^	0.132^b^	<0.001^b^
HDL-C (mmol/L)	1.40 (1.18–1.68)	−0.046^a^	0.189^a^	−0.109^b^	0.002^b^
LDL-C (mmol/L)	2.72 (2.27–3.27)	−0.035^a^	0.318^a^	0.012^b^	0.727^b^
FBG (mmol/L)	4.84 (4.23–5.76)	<0.001^a^	0.995^a^	0.022^b^	0.530^b^

*GFR, glomerular filtration rate; MetS, metabolic syndrome; WC, waist circumference; SBP, systolic blood pressure; DBP, diastolic blood pressure; HDL-C, high-density lipoprotein cholesterol; LDL-C, low-density lipoprotein cholesterol; FBG, fasting blood glucose*.

a *correlation coefficients (r-values) and P-values with serum homocysteine levels*.

b *correlation coefficients (r-values) and P-values with serum uric acid levels*.

c *correlation coefficients (r-values) and P-values with hyperhomocysteinemia*.

d *correlation coefficients (r-values) and P-values with hyperuricemia*.

**Table 2:** Associations of hyperhomocysteinemia and hyperuricemia with GFR < 60 ml/min/1.73 m^2^ and MetS in Chinese centenarians.

**Variables**		**Models**	**EXP (β)**	**95% confidence interval**	***P*-value**
GFR < 60 ml/min/1.73 m^2^	Hyperhomocysteinemia	Crude	7.287	3.289–16.144	<0.001
		Adjusted^a^	7.028	3.142–15.722	<0.001
	Hyperuricemia	Crude	4.625	3.329–6.425	<0.001
		Adjusted^a^	4.710	3.346–6.631	<0.001
MetS	Hyperhomocysteinemia	Crude	2.884	1.030–8.076	0.044
		Adjusted^b^	3.156	1.124–8.862	0.029
	Hyperuricemia	Crude	1.636	1.086–2.466	0.019
		Adjusted^b^	1.677	1.109–2.538	0.014

*GFR, glomerular filtration rate; MetS, metabolic syndrome*.

a *Logistic regression analyses were performed after adjusting for age, sex, waist circumference, systolic blood pressure, diastolic blood pressure, triglyceride, high-density lipoprotein cholesterol, low-density lipoprotein cholesterol, and fasting blood glucose*.

b *Logistic regression analyses were performed after adjusting for age and sex*.

**Table 3:** Associations of serum homocysteine and uric acid levels with GFR and all features of MetS in Chinese centenarians.

**Variables**		**Models**	**Standard β**	**t**	**Standard error**	***P*-value**
GFR (ml/min/1.73 m^2^)	Homocysteine (umol/L)	Crude	−0.356	−10.827	<0.001	<0.001
		Adjusted^a^	−0.330	−10.055	<0.001	<0.001
	Uric acid (umol/L)	Crude	−0.519	−17.238	<0.001	<0.001
		Adjusted^a^	−0.530	−16.986	<0.001	<0.001
WC (cm)	Homocysteine (umol/L)	Crude	0.137	3.933	<0.001	<0.001
		Adjusted^b^	0.134	3.841	<0.001	<0.001
	Uric acid (umol/L)	Crude	0.184	5.301	<0.001	<0.001
		Adjusted^b^	0.170	4.755	<0.001	<0.001
SBP (mmHg)	Homocysteine (umol/L)	Crude	0.051	1.441	<0.001	0.150
		Adjusted^b^	0.060	1.710	<0.001	0.088
	Uric acid (umol/L)	Crude	−0.041	−1.156	<0.001	0.248
		Adjusted^b^	−0.018	−0.486	0.009	0.627
DBP (mmHg)	Homocysteine (umol/L)	Crude	0.034	0.965	<0.001	0.335
		Adjusted^b^	0.038	1.063	0.037	0.288
	Uric acid (umol/L)	Crude	−0.029	−0.832	<0.001	0.406
		Adjusted^b^	−0.018	−0.492	0.005	0.623
Triglyceride (mmol/L)	Homocysteine (umol/L)	Crude	0.091	2.599	0.001	0.010
		Adjusted^b^	0.101	2.875	0.001	0.004
	Uric acid (umol/L)	Crude	0.152	4.354	<0.001	<0.001
		Adjusted^b^	0.195	5.482	<0.001	<0.001
HDL-C (mmol/L)	Homocysteine (umol/L)	Crude	−0.038	−1.093	<0.001	0.275
		Adjusted^b^	−0.031	−0.877	<0.001	0.381
	Uric acid (umol/L)	Crude	−0.120	−3.424	<0.001	0.001
		Adjusted^b^	−0.098	−2.722	<0.001	0.007
LDL-C (mmol/L)	Homocysteine (umol/L)	Crude	−0.022	−0.620	<0.001	0.536
		Adjusted^b^	−0.014	−0.384	<0.001	0.701
	Uric acid (umol/L)	Crude	0.022	0.617	<0.001	0.538
		Adjusted^b^	0.048	1.334	<0.001	0.183
FBG (mmol/L)	Homocysteine (umol/L)	Crude	0.018	0.504	<0.001	0.614
		Adjusted^b^	0.017	0.470	<0.001	0.638
	Uric acid (umol/L)	Crude	−0.003	−0.079	<0.001	0.937
		Adjusted^b^	−0.006	−0.173	<0.001	0.863

*GFR, glomerular filtration rate; MetS, metabolic syndrome; WC, waist circumference; SBP, systolic blood pressure; DBP, diastolic blood pressure; HDL-C, high-density lipoprotein cholesterol; LDL-C, low-density lipoprotein cholesterol; FBG, fasting blood glucose*.

a *linear regression analyses were performed after adjusting for age, sex, waist circumference, systolic blood pressure, diastolic blood pressure, triglyceride, high-density lipoprotein cholesterol, low-density lipoprotein cholesterol, and fasting blood glucose*.

b *linear regression analyses were performed after adjusting for age and sex*.

## Discussion

The MetS is clinically important as it causes a three- to four-fold increased incidence of cardiovascular diseases and worsens survival prognosis by increasing cardiovascular events and mortality (1–4). About one-fifth of the adult population in the United States had high cardiovascular risk, with the prevalence of MetS being estimated at 22.9% (2). Homocysteine is a thiol-containing amino acid that is formed during the conversion of methionine to cysteine (5, 6). As studies have suggested, serum homocysteine levels have been related to MetS in general adults and the elderly population (7, 8). Nevertheless, the relationship between hyperhomocysteinemia and MetS has been a matter of debate (9–11). Moreover, only a limited number of studies have discussed this relationship in China, not to say in Chinese centenarians. The current study indicated a tight connection between hyperhomocysteinemia and MetS, and identified WC and triglyceride as the factors that linked MetS to serum homocysteine levels in Chinese centenarians. Hyperhomocysteinemia may thus, turn out to be a good marker in the development of MetS. Several mechanisms may explain their relationship, and hyperhomocysteinemia may be the cause and/or the consequence of insulin resistance (10). Insulin resistance has been proposed as the main regulator of serum homocysteine levels (33). In previous animal experiments, hyperhomocysteinemia has been found to be a result of hyperinsulinemia (34). Cystathionine-b-synthase, the key enzyme of transsulfuration pathway in homocysteine metabolism, is downregulated in an insulin-resistant state (35). At the same time, elevated levels of serum homocysteine may result in insulin resistance through an inhibition of insulin-receptor kinase activity *in vitro* (36). Hyperhomocysteinemia has been reported before the onset of MetS, and may be the initial signature before the development of MetS (37).

The relationship between hyperuricemia and MetS has been studied before; however, it is yet to be studied in the centenarians, especially in China. Moreover, this relationship remains a debatable topic to be studied in general populations (12, 13). In the current study, hyperuricemia was associated with MetS through WC, triglyceride, and HDL-C. The underlying mechanism is still not well understood, though insulin resistance is suspected to be the mechanism interlinking hyperuricemia with the development of MetS (13). A previous study has proposed that uric acid-mediated upregulation of the adipose renin–angiotensin system may cause insulin resistance (38). Uric acid may also directly contribute to the development of insulin resistance in adipose tissue, possibly through redox modulation or adiponectin (39). Hyperuricemia may indicate MetS, which itself is associated with cardiovascular risk (40). Furthermore, lowering serum levels of uric acid may be a useful strategy for reducing MetS and cardiovascular burden (13).

As a subject that has been previously studied, the relationship between hyperuricemia and renal function has not been confirmed in the centenarians, especially in China (15). Even in general population, previous studies have pointed out disputable results about this relationship (16–18). The current study supported that hyperuricemia significantly interacted with renal function, mediated by WC, triglyceride, and HDL-C. Uric acid, which is a final product of purine metabolism, is mainly eliminated in the urine. Elevated levels of serum uric acid have been considered to be a marker for renal function (41). Furthermore, hyperuricemia may affect renal function through an induction of endothelial dysfunction, inflammatory reaction, and oxidative stress (42, 43). This effect occurs mainly because uric acid stimulates the renin–angiotensin system and inhibits vascular nitric oxide synthesis (44). Experimental studies have also shown that hyperuricemia induces the development of glomerular arteriolopathy that impairs renal autoregulation and causes glomerular hypertension, leading eventually to glomerulosclerosis and interstitial fibrosis (45). It will probably be necessary to control uric acid levels so as to improve renal function.

In previous studies, hyperhomocysteinemia has been found to have a positive correlation with renal function (19). However, the number of studies analyzing this correlation is limited and their results are contradicting, especially in Chinese centenarians (20). The current study illustrated that hyperhomocysteinemia was significantly associated with renal function through WC and triglyceride. Renal function decline may not only reduce renal extraction of homocysteine due to decreasing plasma flow, but also affect homocysteine metabolism through homocysteine remethylation, one of the main metabolitic pathways in homocysteine degradation (46, 47). Meanwhile, hyperhomocysteinemia has been suggested as an important pathogenic factor leading to glomerular injury, dysfunction, and sclerosis (48). Increased oxidative stress and decreased antioxidant defense function, caused by hyperhomocysteinemia, have been proved to be associated with renal function (21).

The current study had several limitations. Firstly, several parameters, such as a specific diet, vitamin B12, and mental status, were difficult to be obtained in this epidemiological study. These parameters needed to be evaluated with great complications by nutritionists and psychiatrists. Secondly, there was no evidence for a causal relationship provided and no correlation with mortality tested in the current study. Thirdly, only data from centenarians were analyzed in the current study, and there was no comparison with the elderly aged < 100 years. Future studies should be performed to not only compare these centenarians with a cohort of the elderly aged < 100 years from the same region, but also provide evidence for a causal relationship and test the correlation with mortality in the following years.

## Conclusion

As the first in the world, the current study demonstrated that both hyperhomocysteinemia and hyperuricemia had important relationships with MetS and renal function in Chinese centenarians. Hyperuricemia and hyperhomocysteinemia that could help identify, while also affecting, the development of MetS and renal dysfunction may unfold complex relationships between MetS, renal function, and cardiovascular risk, and provide effective prevention strategies for these conditions.

## Author contributions

SF, YY, YZ, and FL designed the study, collected and analyzed the data, and wrote the paper.

### Conflict of interest statement

The authors declare that the research was conducted in the absence of any commercial or financial relationships that could be construed as a potential conflict of interest.
